# Factors influencing knowledge about childhood autism among final year undergraduate Medical, Nursing and Psychology students of University of Nigeria, Enugu State, Nigeria

**DOI:** 10.1186/1824-7288-36-44

**Published:** 2010-06-13

**Authors:** Monday N Igwe, Muideen O Bakare, Ahamefule O Agomoh, Gabriel M Onyeama, Kevin O Okonkwo

**Affiliations:** 1 Department of Psychological Medicine, University of Nigeria Teaching Hospital Enugu, Nigeria; 2 Child and Adolescent Unit, Federal Neuropsychiatric Hospital, New Haven, Enugu, Nigeria

## Abstract

**Background:**

Knowledge and awareness about childhood autism is low among health care workers and the general populace in Nigeria. Poor knowledge about childhood autism among final year medical, nursing and psychology students who would form tomorrow's child health care professionals can compromise early recognition and interventions that are known to improve prognosis in childhood autism. Educational factors that could be influencing knowledge about childhood autism among these future health care professionals are unknown. This study assessed knowledge about childhood autism among final year undergraduate medical, nursing and psychology students in south-eastern Nigeria and determined the factors that could be influencing such knowledge.

**Methods:**

One hundred final year undergraduate students were randomly selected from each of the Departments of Medicine, Nursing Science and Psychology respectively of University of Nigeria, Enugu State, Nigeria making a sample size of three hundred. A socio-demographic questionnaire and knowledge about childhood autism among health workers (KCAHW) questionnaire were administered to the students.

**Results:**

The total mean score for the three groups of students on the KCAHW questionnaire was 10.67 ± 3.73 out of a possible total score of 19, with medical, nursing and psychology students having total mean scores of 12.24 ± 3.24, 10.76 ± 3.50 and 9.01 ± 3.76 respectively. The mean scores for the three groups showed statistically significant difference for domain 1 (p = 0.000), domain 3 (p = 0.029), domain 4 (p = 0.000) and total score (p = 0.000), with medical students more likely to recognise symptoms and signs of autism compared to nursing and psychology students. The mean score in domain 2 did not show statistically significant difference among the three groups (p = 0.769). The total score on the KCAHW questionnaire is positively correlated with the number of weeks of posting in psychiatry (r = 0.319, p = 0.000) and the number of weeks of posting in paediatrics (r = 0.372, p = 0.000). The total score is also positively correlated with the number of credit hours of lectures in psychiatry/abnormal psychology (r = 0.324, p = 0.000) and the number of credit hours of lectures in paediatrics (r = 0.372, p = 0.000). The field of study also influenced knowledge about childhood autism (p = 0.000).

**Conclusion:**

Peculiar situation in this environment as signified by inadequate human resources needed in the area of clinical psychology training often times necessitates employing first degree graduates in psychology into clinical positions. This calls for additional exposure of the undergraduate psychology students to training curriculum aimed at improving their early recognition of symptoms of autism spectrum disorders in this environment.

## Background

Childhood autism was first described by Leo Kanner in his classical paper titled 'Autistic disturbances of affective contact' published in 1943 [1]. Since then, awareness, knowledge and further research about this condition have continued to expand globally with observations from several surveys indicating increasing prevalence [2-4]. Whereas there is increasing awareness and research on childhood autism and other pervasive developmental disorders in many parts of the world, knowledge and research about these conditions are at lower ebb in sub-Saharan Africa [5]. It may have been because of this that the occurrence of childhood autism in sub-Saharan Africa was brought to question [6]. There is evidence that childhood autism and other developmental disorders are prevalent in sub-Saharan African countries [7-9].

Njenga [10] had noted that discrimination and lack of access to education and justice are major challenges facing individuals with intellectual disability including children with developmental disorders like childhood autism in Africa. There is need for more policy attention to be focused on intellectual disability and childhood autism in Africa. The need to recognise these children and institute early interventions is essential.

Appreciating the importance of early recognition and intervention for children with autism with regards to a better prognosis, Shah [11] evaluated the level of awareness about childhood autism among medical students at different stages of their training. He observed that fourth-year students were significantly more likely to respond correctly to questions related to diagnostic criteria and core symptoms. There were no significant differences for other aspects such as possible causes, intelligence quotient profiles, prognosis and treatment. Based on this, he went ahead to suggest that more emphasis needs to be placed on teaching medical students about autism if diagnosis and access to intervention are to be improved.

Physicians' knowledge about childhood autism greatly influences the average age of diagnosis of autistic spectrum disorders and such knowledge or lack of it may actually be a reflection of training received by such physicians [12].

Heidgerken et al [13] assessed knowledge about diagnostic criteria, course, treatment and prognosis of autism among professionals that included psychiatrists, paediatricians, primary health care providers and clinical psychologists working at the Center for Autism Related Disabilities (CARD). They noticed display of accurate knowledge of DSM-IV criteria for diagnosis but significant variations between the specialists and primary health care providers on prognosis, course and treatment of autism.

Schwartz & Drager [14] noted that most of the school based speech language pathologists practising in thirty three states of United States of America had accurate knowledge about the clinical presentation of children with autism but had mixed perceptions of diagnostic criteria for autism, even though most of them had access to information on autism at some level of their educational training. Some of them lacked confidence in their abilities to provide services to children with autism and indicated in their responses that they could have benefitted from additional training in the area of autism.

The African Network for the Prevention and Protection against Child Abuse and Neglect (ANPPCAN), Nigerian chapter in collaboration with the World Bank in 2007 carried out a survey aimed at assessing the level of awareness on autism among the general public and health care workers in Enugu State, South-Eastern Nigeria. The survey showed a very low level of awareness among the general public and a low to moderate level of knowledge among various categories of health care workers. The highest level of knowledge was found among health care professionals working in psychiatric facilities within the region [15].

Management of childhood autism and other pervasive developmental disorders requires services of professionals like psychiatrists, paediatricians and psychologists among others [2]. Undergraduate final year medical, nursing and psychology students after their training will form members of such multidisciplinary teams. It is possible that the low level of knowledge about childhood autism among health care workers documented earlier in this environment [5,15,16] may be due to insufficient exposure to adequate lectures and postings in paediatrics, psychiatry and psychology during training.

Therefore, there is the need to assess the level of knowledge about childhood autism among final year undergraduate medical, nursing and psychology students who shortly after training may form part of the multidisciplinary team that would be caring for children with autism and other developmental disorders.

This study is aimed at assessing knowledge about childhood autism and evaluating factors that influence such knowledge among undergraduate final year medical, nursing and psychology students of University of Nigeria, Enugu State, Nigeria.

## Methods

### Location

Location of the study was at the University of Nigeria, Enugu State, Nigeria. It is one of the first generation universities in Nigeria, having been established in 1960. The institution runs both undergraduate and postgraduate programmes. The Departments of Medicine and Nursing Science are sited at Enugu campus of the University while the Department of Psychology is located at Nsukka campus in Enugu state, Nigeria. The Department of Medicine offers a six year programme leading to an award of the Bachelor of Medicine, Bachelor of Surgery. The Bachelor of Science degree in Psychology requires four years of study. The Bachelor of Science degree in Psychology is not regarded as a clinical qualification in Nigeria. However, because of the limited facilities for training postgraduate clinical psychologists and their very few numbers in meeting healthcare demands, many graduate degree holders are often employed into clinical posts to fill the unmet gaps in demand in Nigeria. These graduate degree holders are therefore faced with challenges of discharging clinical responsibilities. The Bachelor of Science in Nursing takes a period of five years to complete.

### Ethical approval

The ethical approval for the study was obtained from the Institutional Review Board (IRB) of Federal Neuro-psychiatric Hospital, New Haven, Enugu, Enugu State, Nigeria. Written informed consent was also obtained from the students that participated in the study.

### Participants and sampling method

The participants involved in the study were final year undergraduate medical, nursing and psychology students of the University of Nigeria. One hundred students who have done all relevant postings and lectures as prescribed in the academic curriculum in paediatrics, psychiatry and psychology were randomly selected from each final year class of the Departments of Medicine, Nursing and Psychology making a total sample size of three hundred.

## Materials

### Socio-demographic questionnaire

A socio-demographic questionnaire was used to obtain demographic information like gender, age, marital status, religion, ethnicity, field of study, duration of postings in psychiatry and paediatrics, number of credit hours of lectures in paediatrics, psychiatry, child and adolescent psychology.

### Knowledge about childhood autism among health workers (KCAHW) questionnaire [5]

The KCAHW is a self administered questionnaire that contains a total of nineteen (19) - item question. It has been established to have good test-retest reliability with good overall internal consistency [5]. Each of the items throughout has three (3) options to choose from with only one of these three options being correct. The correct option on each item attracts a score of one (1), while the other two options that are incorrect attract a score of zero (0) each.

The KCAHW questionnaire is divided into the following four (4) domains:

#### Domain 1

Contains eight (8) items that address the impairments in social interaction usually found in children with childhood autism. A maximum and minimum score of 8 and 0 respectively are possible in this domain.

#### Domain 2

Contains only one (1) item that addresses impairment in the area of communication and language development, which is part of the symptom presentation in children with childhood autism. A maximum and minimum score of 1 and 0 respectively are possible in this domain.

#### Domain 3

Contains four (4) items that address the area of obsessive and compulsive pattern of behaviour found in children with childhood autism, a pattern of behaviour which had been described as restricted, repetitive and stereotyped. A maximum and minimum score of 4 and 0 respectively are possible in this domain.

#### Domain 4

Contains six (6) items that address knowledge on what type of disorder childhood autism is, possible co-morbid conditions and onset of childhood autism in affected children. A maximum and minimum score of 6 and 0 respectively are possible in this domain.

A maximum and minimum total score of 19 and 0 respectively are possible when the four domain scores are added together. The content of the questionnaire and the scoring system are shown in Appendix 1. The mean total score on the KCAHW questionnaire among a particular sample population is a measure of level of knowledge about childhood autism among that particular population. A total score of 19, which is the maximum score possible on the KCAHW questionnaire indicates adequate knowledge of symptoms and signs that characterize autism. This adequate knowledge may enhance early recognition, diagnosis, appropriate referral and interventions that are known to improve prognosis in children with childhood autism and other pervasive developmental disorders.

### Procedure

The socio-demographic and KCAHW questionnaire were administered to the three hundred students (one hundred from each of the final year undergraduate classes of the three departments). The questionnaires were completed and collected immediately from respondents. This was to avoid consultation of study materials or discussion with colleagues which could have influenced the students' responses to the questions on KCAHW questionnaire.

### Data analysis

The data were analyzed using Statistical Package for Social Sciences (SPSS), version 16. The mean scores in each domain and the mean total scores were calculated for each group of students in the three departments. The mean total scores were related to the socio-demographic variables of the students using one way analysis of variance (ANOVA) and Spearman's Rho correlation statistics.

## Results

A total of three hundred (300) final year undergraduate students participated in the study, one hundred each from the Departments of Medicine, Nursing Science and Psychology. There were sixty two (62%) male and thirty eight (38%) female medical students, sixteen (16%) male and eighty four (84%) female nursing students while fifty nine (59%) male and forty one (41%) female were psychology students. The mean ages of the medical, nursing and psychology students were 25.4 ± 2.46 years, 25.67 ± 3.79 years and 24.39 ± 2.35 years respectively. The total mean age was 25.15 ± 2.98 years. Table 1 shows the socio-demographic variables of the participants.

**Table 1:** Socio-demographic variables of the students

Socio-demographic variables	Medicine	Nursing	Psychology
**Gender**			
Male	62 (62%)	16 (16%)	59 (59%)
Female	38 (38%)	84 (85%)	42 (41%)
**Age (years)**			
Mean ± SD	25.40 ± 2.46	25.67 ± 3.79	24.39 ± 2.35
**Marital status**			
Single	95 (95%)	74 (74%)	93 (5%)
Married	5 (5%)	26 (26%)	6 (6%)
Divorced/separated	0 (0%)	0 (0%)	1 (1%)
**Ethnic group**			
Igbo	96 (96%)	97 (97%)	99 (99%)
Others	4 (4%)	3 (3%)	1 (1%)
**Duration of clinical postings (weeks)**	4	4	1
Psychiatry	16	4	0
Paediatrics			
**Number of credit hours of lectures (semester)**			
Psychiatry/Abnormal psychology	4	2	2
Paediatrics	8	2	0
Child psychology	1	1	2

### Pattern of distribution of scores on the KCAHW questionnaire among the students

The total mean score on the KCAHW questionnaire among the students that participated in the study was 10.67 ± 3.73 out of a total of 19 possible. Mean score for the medical students was 12.24 ± 3.24 while nursing and psychology students had mean scores of 10.76 ± 3.50 and 9.01 ± 3.76 respectively. The mean scores in Domain 1, which deals with questions in the area of impairments in social interaction found in childhood autism, were 5.97 ± 1.98 for the medical students, 5.24 ± 2.06 for nursing science students and 4.09 ± 1.99 for psychology students. The mean scores in Domain 2 which deals with a question on communication impairments that often characterized childhood autism were 0.63 ± 0.49, 0.60 ± 0.49, 0.58 ± 0.50 for medical, nursing and psychology students respectively. Domain 3, which deals with questions on obsessive and repetitive behavioural pattern that often characterized childhood autism, yielded mean scores of 2.21 ± 1.30 for medical students, 1.77 ± 1.17 for nursing students and 1.84 ± 1.28 for psychology students. Domain 4 that addressed questions on what type of disorder childhood autism is and possible associated co-morbidity recorded the mean scores of 3.43 ± 1.27, 3.16 ± 1.30 and 2.49 ± 1.37 for medical, nursing and psychology students respectively.

The mean score for the three groups showed statistically significant difference for Domain 1 (p = 0.000), domain 3 (p = 0.029), domain 4 (p = 0.000) and total score (p = 0.000), with medical students more likely to recognise symptoms and signs of autism followed by nursing and psychology students. The mean score in domain 2 did not show statistically significant difference among the three groups (p = 0.769).

### Factors influencing knowledge about childhood autism among final year undergraduate Medical, Nursing and Psychology students

The total score on KCAHW questionnaire was positively correlated with number of weeks of posting in psychiatry (r = 0.319, p = 0.000). Medical and nursing students who spent four weeks each in psychiatric facilities doing clinical postings were found to be more likely to recognise signs and symptoms of childhood autism compared to psychology students who spent only one week of posting in psychiatric facilities.

The total score on the KCAHW questionnaire was also positively correlated with the number of weeks of posting in paediatrics (r = 0.372, p = 0.000). Medical students spent eight weeks in junior paediatrics and eight weeks in senior paediatric posting making a total of sixteen weeks. Nursing students spent only four weeks of posting in paediatrics while psychology students did not have any exposure to paediatrics.

The total score on the KCAHW questionnaire was positively correlated with the number of credit hours of lectures in psychiatry/abnormal psychology (r = 0.324, p = 0.000). Medical students received a cumulative four credit hours of lectures in psychiatry/abnormal psychology for a semester during their training, while nursing and psychology students had two credit hours of lectures respectively for a semester.

Total score on the KCAHW questionnaire was also positively correlated with the number of credit hours of lectures in paediatrics (r = 0.372, p = 0.000). Medical students received a cumulative eight credit hours of lectures in paediatrics during their training while nursing students get two credit hours of lectures taught as maternal and child health. Psychology students received no formal lectures in paediatrics.

The field of study influenced total score on KCAHW questionnaire among the three groups of students and the influence is statistically significant (p = 0.000). Medical students had mean total score of 12.24 ± 3.21 as against 10.76 ± 3.50 and 9.01 ± 3.76 for nursing and psychology students respectively out of a total possible score of 19.

## Discussion

The total mean score on the KCAHW questionnaire among the 3 groups studied was 10.67 ± 3.73. This revealed a lower level of knowledge about childhood autism compared to the mean score of 12.35 ± 4.40 obtained among health care workers who are practising nurses in our earlier study [16]. The possible explanation would be that this group of nurses were equipped with relatively more knowledge about childhood autism as a result of their working experience.

In this study, medical students had the highest total mean score, followed by nursing students and then psychology students had the lowest score. In the academic curricula of these students, medical students have the longest duration of posting exposure in paediatrics (sixteen weeks) as compared to nursing students (four weeks) and psychology students who have no period of posting in paediatrics (zero week). The duration of posting in psychiatry also differs among the three groups of students with medical and nursing students having a period of four weeks each as against one week for psychology students.

The number of credit hours of lectures for medical students was also highest in paediatrics, followed by nursing students while psychology students had no exposure. The number of credit hours of lectures in psychiatry/abnormal psychology was also highest for medical students, followed by nursing students and psychology students who have the same number of credit hours of lectures.

A significant difference was found between mean scores on the KCAHW questionnaire and area of specialties, with medical students more likely to score highest followed by nursing students and then psychology students scoring the lowest, though graduate degree qualification in psychology is not regarded as a clinical qualification. The longer exposure in lectures and postings influencing knowledge about childhood autism, as observed in this study concurs with earlier findings by Shah [11].

The management of children with autism requires a multi-disciplinary approach requiring services of professionals like paediatricians, nurses and psychologists among others. The discrepancy in knowledge found among the students of these various specialties of medicine, nursing and psychology in our study is likely to be due to the variations in periods of postings and hours of lectures allocated to paediatrics, psychiatry and psychology during the undergraduate training period of these students.

This study demonstrates that knowledge about childhood autism among final year undergraduate medical, nursing and psychology students in Enugu, Nigeria is low. This is in keeping with the low level of knowledge and awareness among health workers and the general populace in south-eastern Nigeria which had earlier been documented [5,15,16].

In view of the need to give adequate attention to childhood autism and other developmental disorders in Sub-Saharan African countries [10], some basic clinical exposures may need to be incorporated into the curriculum of undergraduate psychology training in this environment.

These final year students following graduation, in addition to being the first to come in contact with children suffering from various childhood developmental disorders, would equally be members of the multidisciplinary team that would be managing these children in the near future. Thus, the need to assess baseline knowledge of trainees in these three academic areas, so as to ensure proper planning aimed at improving their clinical ability to recognise children with symptoms of autism spectrum disorders in this environment.

## Conclusion

Peculiar situation in this environment as signified by inadequate human resources needed in the area of clinical psychology training often times necessitates employing first degree graduates in psychology into clinical positions. This indicates a need for additional exposure of the undergraduate psychology students to training curriculum aimed at improving their early recognition of symptoms of autism spectrum disorders in this environment.

## Competing interests

The authors declare that they have no competing interests.

## Authors' contributions

MNI was involved in collection of data and writing the initial draft of the manuscript. MOB was involved with data analysis and writing the initial draft of the manuscript. All authors contributed to the conception of the study and were involved in writing and revising the manuscript. All authors read and approved the final draft of the manuscript.

## Appendix 1

### Knowledge about Childhood Autism among Health Workers (KCAHW) Questionnaire

Please do not consult formal text books to answer these questions.

Thank you for your co-operation.

The following behaviours best describe a child with childhood autism:

#### Domain 1

i. Marked impairment in use of multiple non-verbal behaviours such as eye to eye contact, facial expression, body postures and gestures during social interaction?

(A) Don't Know, (B) Yes, (C) No

ii. Failure to develop peer relationship appropriate for developmental age?

(A) Don't Know, (B) Yes, (C) No

iii. Lack of spontaneous will to share enjoyment, interest or activities with other people? (A) Don't Know, (B) Yes (C) No

iv. Lack of social or emotional reciprocity? (A) Don't Know (B) Yes, (C) No

v. Staring into open space and not focusing on anything specific? (A)Don't Know, (B) Yes, (C) No

vi. The child can appear as if deaf or dumb? (A) Don't Know (B) Yes, (C) No

vii. Loss of interest in the environment and surroundings?

(A) Don't Know, (B) Yes, (C) No

viii. Social smile is usually absent in a child with Autism?

(A)Don't Know, (B) Yes (C) No

#### Domain 2

i. Delay or total lack of development of spoken language?

(A) Don't Know (B) Yes (C) No

#### Domain 3

i. Stereotyped and repetitive movement (e.g. Hand or finger flapping or twisting)?

(A) Don't Know (B) Yes, (C) No

ii. May be associated with abnormal eating habit?

(A) Don't Know, (B) Yes, (C) No

iii. Persistent preoccupation with parts of objects?

(A) Don't Know (B) Yes, (C) No

iv. Love for regimented routine activities? (A) Don't Know (B) Yes, (C) No

#### Domain 4

i. Autism is Childhood Schizophrenia? (A) Don't Know (B) Yes (C) No

ii. Autism is an auto-immune condition? (A) Don't Know (B) Yes (C) No

iii. Autism is a neuro-developmental disorder? (A) Don't Know (B) Yes (C) No

iv. Autism could be associated with Mental Retardation? (A) Don't Know (B) Yes (C) No

v. Autism could be associated with Epilepsy? (A) Don't Know (B) Yes (C) No

vi. Onset of Autism is usually in, (A) Neonatal age, (B) Infancy, (C) Childhood

### Scoring of Knowledge about Childhood Autism among Health Workers (KCAHW) questionnaire

#### Domain 1

i Marked impairment in use of multiple non-verbal behaviours such as eye to eye contact, facial expression, body postures and gestures during social interaction?

(A) 0 (B) 1 (C) 0

ii Failure to develop peer relationship appropriate for developmental age? (A) 0 (B) 1 (C) 0

iii. Lack of spontaneous will to share enjoyment, interest or activities with other people?

(A) 0 (B) 1 (C) 0

iv Lack of social or emotional reciprocity? (A) 0 (B) 1 (C)

v Starring into open space and not focusing on anything specific?

(A) 0 (B) 1 (C) 0

vi. The child can appear as if deaf or dumb? (A) 0 (B) 1 (C) 0

vii. Loss of interest in the environment and surroundings? (A) 0 (B) 1 (C) 0

viii. Social smile is usually absent in a child with Autism? (A) 0 (B) 1 (C) 0

#### Domain 2

i. Delay or total lack of development of spoken language? (A) 0 (B) 1 (C) 0

#### Domain 3

i. Stereotyped and repetitive movement (e.g. Hand or finger flapping or twisting)?

(A) 0 (B) 1 (C) 0

ii. May be associated with abnormal eating habit? (A) 0 (B) 1 (C) 0

iii. Persistent preoccupation with parts of objects? (A) 0 (B) 1 (C) 0

iv. Love for regimented routine activities? (A) 0 (B) 1 (C) 0

#### Domain 4

i. Autism is Childhood Schizophrenia? (A) 0 (B) 0 (C) 1

ii. Autism is an auto-immune condition? (A) 0 (B) 0 (C) 1

iii Autism is a neuro-developmental disorder? (A) 0 (B) 1 (C) 0

iv. Autism could be associated with Mental Retardation? (A) 0 (B) 1 (C) 0

v. Autism could be associated with Epilepsy? (A) 0 (B) 1 (C) 0

vi Onset of Autism is usually in, (A) 0 (B) 0 (C) 1

A total maximum score of 19 and a minimum score of 0 are possible. The average score on the KCAHW questionnaire among a particular sample population gives an index level of knowledge about childhood autism in that particular population.
